# Chimney grafts in renal arteries: a clinical model for coronary perfusion in future transcatheter aortic root repair techniques

**DOI:** 10.1186/s13019-020-01184-1

**Published:** 2020-06-09

**Authors:** Enrico Ferrari, Changtian Wang, Denis Berdajs, Ludwig Karl von Segesser

**Affiliations:** 1 Cardiac Surgery, Cardiocentro Ticino, Via Tesserete 48, 6900 Lugano, Switzerland; 2 Cardiovascular Surgery, University Hospital of Zurich, Zurich, Switzerland; 3 Cardiovascular Surgery, Nanjing Jinling Hospital, Nanjing, China; 4 Cardiovascular Surgery, University Hospital of Basel, Basel, Switzerland; 5 Cardiovascular Research Unit, University Hospital of Lausanne, Lausanne, Switzerland

**Keywords:** Chimney graft technique, Endovascular aorta repair, Transcatheter aortic root replacement

## Abstract

**Objectives:**

Given the similarities between coronary ostia and renal arteries, chimney grafts (CG) for kidney perfusion during abdominal endovascular aneurysm repair (EVAR) can be considered for coronary perfusion in future transcatheter aortic root repair (TARR) techniques. We analysed the results of renal CG and compared anatomic and technical details with root and coronary anthropometric data.

**Methods:**

Current status of kidney perfusion with CG was reviewed from literature. Anatomic details, technical data, CG performance and clinical outcome were collected and analysed. Anatomic details of aortic landing zone and renal arteries were compared with human anthropometric data of aortic root, ascending aorta and coronary ostia.

**Results:**

Seventeen articles reported 430 patients (mean age:74.5 ± 2.9 years) treated with renal CG. Mean length and diameter of proximal landing zone were 2.0 ± 2.0 mm and 26.4 ± 4.3 mm, respectively (anthropometric correspondence: ascending aorta diameter of 29.3 mm). Aortic endograft mean diameter was 26.4 ± 7.3 mm with reported oversize of 19.5 ± 6.0%. In total, 590 renal arteries were treated (left:325; right:265; bilateral:139 cases). Mean left and right renal artery diameters were 5.7 ± 0.6 mm and 5.8 ± 0.7 mm, respectively (anthropometric correspondence: coronary ostia diameters of 4.8 mm (left) and 3.7 mm (right)) with reported CG oversize of 19.75 ± 6% (left) and 18.1 ± 5.1% (right). Mean follow-up time was 16.5 ± 8.5 months, CG occlusion rate was 3.2% and endoleak I or II was reported in 83 patients (19.3%), requiring 7 procedures.

**Conclusions:**

CG provides satisfactory results in patients with suitable renal artery diameter. Based on aortic root and coronary anthropometric data, CG can be considered in future TARR technologies for coronary perfusion but further tests for flow evaluations are mandatory.

## Introduction

Recent reports showed that endovascular techniques for non-dissected ascending aorta diseases provide a period of stable conditions followed by new plans of more definitive treatments. In a review of 67 high-risk patients, endografts were mainly located in the ascending aorta (zone-0), above the sino-tubular junction, and provided satisfactory outcomes [1]. However, when the root is severely dilated, the valve is diseased and the patient is at high-risk for surgery, commercially available endoprosthesis and transcatheter valves are not fully effective. Therefore, a new concept of composite-graft (valved-endoprosthesis) for transcatheter aortic root repair (TARR) in patients at risk is desirable but the big challenge for the development of this technology is the way the coronaries are perfused.

Originally described by Greenberg et al., chimney grafts (CG) were firstly employed as bailout procedures during abdominal aorta endovascular aneurysm repairs (EVAR) and balloon-expanding stents were used to preserve the renal flow after the endoprosthesis deployment [2]. Results from the PERICLES registry (898 renal CG in 517 patients) showed a success rate of 97%, mortality of 3.6%, and a CG patency of 94% at 17-month follow-up time [3]. They concluded that CG provide a safe and effective “off-the-shelf” solution in complex EVAR procedures. Consequently, while developing TARR prototypes we realized that CG can also be considered for the coronary perfusion in TARR, being the departure of the coronary arteries anatomically similar to the renal arteries. In order to verify the adaptability of CG to TARR we (1) reviewed the available literature on renal CG during EVAR, (2) investigated anatomic landmarks, technical details and outcome of renal CG, and (3) compared anatomic data of the descending aorta to anthropometric data of the aortic root.

## Methods

In order to identify published articles describing renal CG during EVAR, we searched in MEDLINE up to June 2019 using medical subject headings (MeSH) and text words supplemented by scanning the bibliographies of recovered articles including “chimney stent grafts”, “chimney technique”, “chimney graft”, “chimney stent”, “chimney”, “snorkel”, “chimney EVAR”, “renal artery” and using the Boolean operator “AND”. Language was limited to articles written in English. All included studies were independently assessed and critically evaluated by three authors (EF, CW, LVS). Differences were resolved in consensus discussions.

### Inclusion criteria

We focused on the presence of renal chimney grafts during abdominal EVAR procedures. Articles that stated the following items were included:

1. renal vascularization achieved by CG implantation during endovascular treatment of an abdominal aortic disease;
2. demographic data;
3. clinical outcomes, mainly including CG patency, endoleak (EL), re-intervention, and follow-up.

To avoid duplicate data, only the most recent report from each centre was accepted for this study.

### Exclusion criteria

Clinical studies with mixed populations and target arteries in which we could not extract definite data about the perfusion of renal arteries with CG during EVAR were excluded from the analysis because accurate information about renal CG performances would have hardly be determined. Case reports, abstracts, correspondence, expert opinions and review articles were not included. We excluded articles describing the “periscope” technique for renal revascularization, as well as articles not providing patients outcome and follow-up.

### Data extraction

We collected the following data: number of patients; gender; mean age; body mass index (BMI); landing zone diameter and length (proximal neck); size and length of the aortic endoprosthesis; oversize of the aortic endoprosthesis (%); right and left renal artery diameter; right and left CG length and diameter; right and left CG oversize (%). We also recorded follow-up length, CG occlusion rate, presence and type of early (< 30 days) or late (> 30 days) endoleak, and type of vascular/endovascular reintervention. Some articles mixed the chimney graft technique with other procedures, such as fenestrated EVAR or open vascular surgery: from those reports, we only extracted data about renal CG (if described). If data could not be clearly extracted, the article was excluded.

### Data analysis

**Fig. 1:**
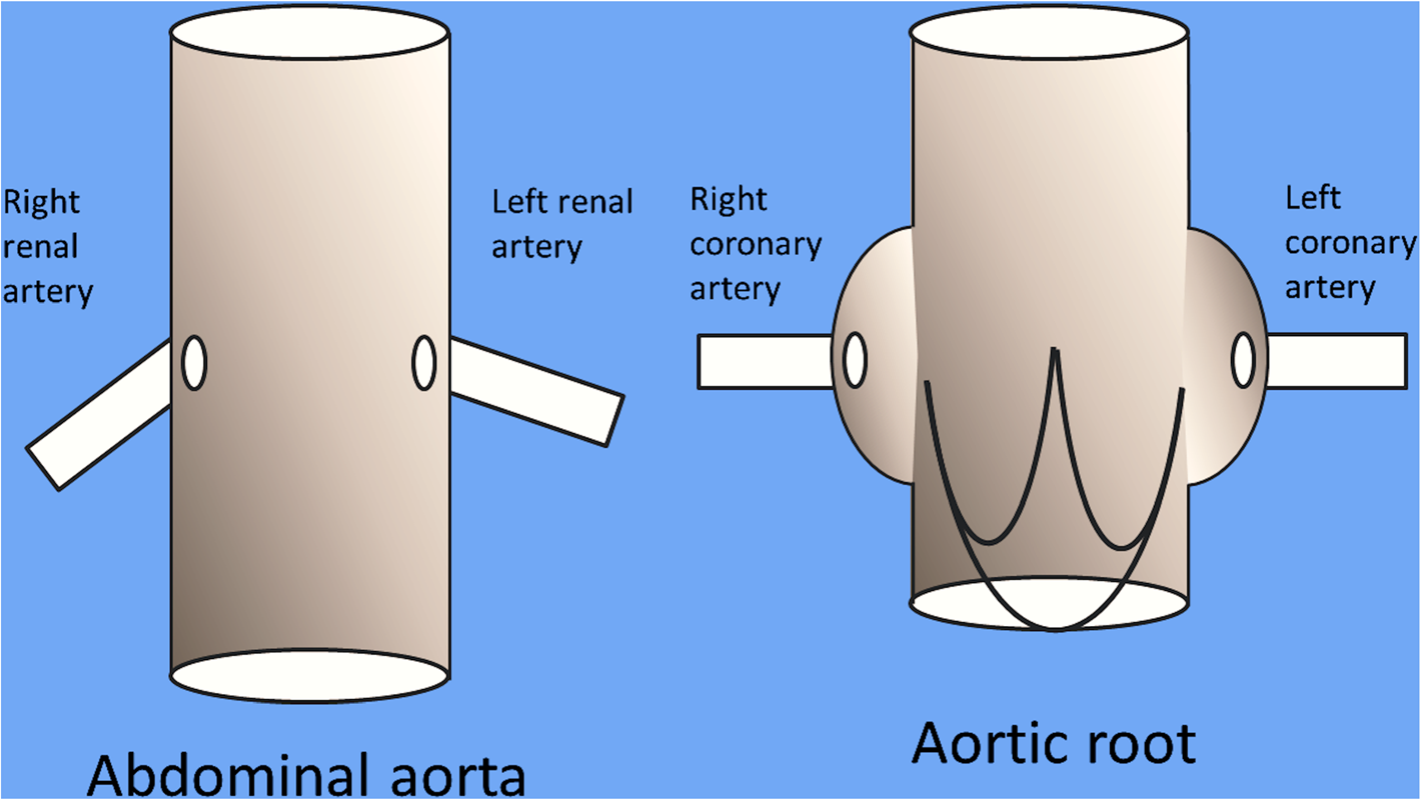
Schematic view of abdominal aorta with renal arteries (left) and aortic root with coronary ostia (right)

Despite flow dynamic in renal arteries differs from flow dynamic in coronary arteries (being the coronary bloodstream mainly guarantee during diastole), the anatomy of the departure of the renal arteries from the abdominal aorta is similar to the departure of the coronary arteries form the ostia in the aortic root: two (almost) opposite collateral side branches departing from a great vessel (Fig. 1). However, attack angle, length and diameter of renal and coronary arteries can variate and, therefore, in order to better consider renal CG as a clinical model for the use of this technique in TARR, we compared anatomic details of patients with renal CG to standard anthropometric data of aortic root and ascending aorta extracted from the available literature and collected in a previous report [4].

### TARR technique

**Fig. 2:**
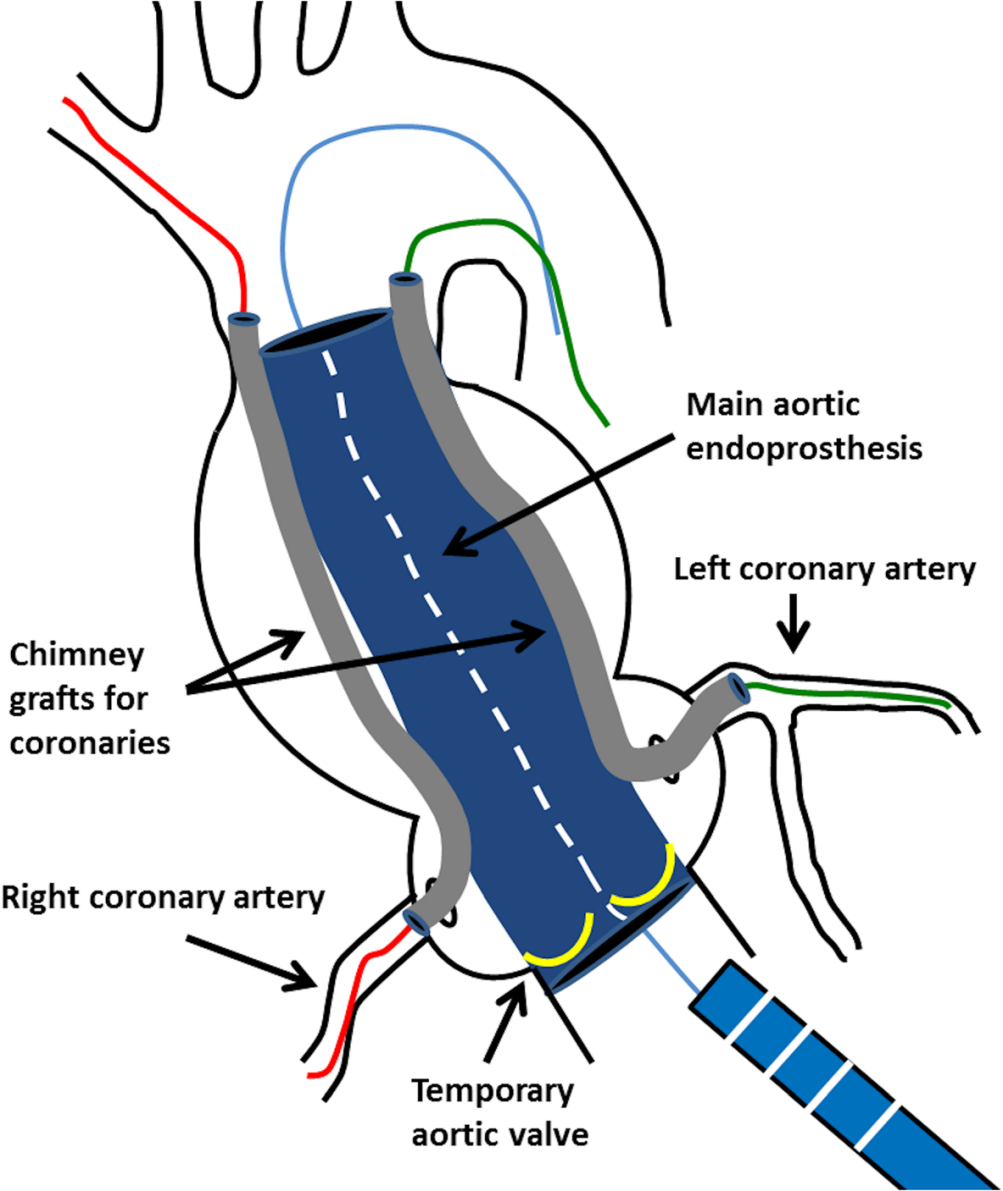
Schematic view of a possible transcatheter aortic root replacement (TARR) technique

Following our preliminary studies in laboratory and in order to support our thesis of CG use in TARR we must consider the following. Main components of endovascular TARR device would be 3: 1) a main aortic endoprosthesis placed from the left ventricle outflow tract (LVOT) (proximal landing zone) to the distal ascending aorta, before the departure of the brachiocephalic trunk (distal landing zone); 2) a temporary aortic valve placed on the ventricular side of the main endograft (not a biological valve); 3) two CG placed into the first part (1–1.5cn) of the coronary artery (ostia) and along the aortic endoprosthesis that guarantee the coronary perfusion (Fig. 2). In a second moment, a clinically available transcatheter aortic valve is placed, transapically or transfemorally, into the main aortic endograft, replacing the temporary valve.

### Statistical analysis

Data were collected and organized in an Apple Numbers spreadsheet (Apple, Cupertino, CA. Version 6.6.2). Descriptive statistic was applied to data collected from the literature. Continuous data are expressed as mean and standard deviation (SD). Dichotomic variables are expressed as numbers with percentage (%).

## Results

Seventeen articles published in 2010–2017 were identified and reviewed [5–21]. A total number of 430 patients (327-males; 93-females; 10-undisclosed) with mean age of 74.5 ± 2.9 years were treated for abdominal aorta disease using EVAR with renal CG. Two papers reported mean BMI of 27.5 ± 9.5 and 26 ± 7, while another paper reported four patients with BMI above 30 [5, 7, 12]. The treated aortic diseases were: juxtarenal aneurysm, abdominal aortic aneurysm, failed EVAR, pseudoaneurysm, abdominal aortic stenosis or occlusion. Mean follow-up time was 16.5 ± 8.5 months (Table 1).

**Table 1:** Patients characteristics from published EVAR series with renal CG

Reference	Patients	Mean age (years)	Gender	Body Mass Index	Aortic disease	Follow-up (months)
Ullery BW 2017 [5]	15	75.8 ± 7.7	12 M; 3F	27.5 ± 9.5	AAA	25.5
Sugimoto M 2017 [6]	38	75 ± 8.1	34 M; 4F	–	AAA	12
Caradu C 2017 [7]	26	74.7 ± 6.9	24 M; 2F	4 with BMI > 30	composite	20 (range:1–35)
Youssef M 2017 [8]	10	–	–	–	failed EVAR	8 (range:3–24)
De Bruin JL 2016 [9]	28	75 (range:60–87)	22 M; 6F	–	JAA	4
Ronchey S 2015 [10]	20	76.1 ± 4.6	16 M; 4F	–	AAA	30.9 ± 20.6 (range:5–58)
XiaoHui M 2015 [11]	42	71 ± 7.0	35 M; 7F	–	JAA	26 ± 13 (range:6–64)
Scali ST 2014 [12]	41	73 ± 8.0	27 M; 14F	26 ± 7	JAA; AAA; failed EVAR	18.2 (range:1.4–41.5)
Ducasse E 2014 [13]	22	73 (range:63–88)	21 M; 1F	–	JAA	18 (range:7–35)
Tolenaar JL 2013 [14]	12	77.2 ± 6.2	11 M; 1F	–	AAA; failed EVAR	10.8 (range:7.4–19.4)
Bin Jabr A 2013 [15]	10	68 ± 4.7	0 M; 10F	–	aortic stenosis or occlusion	33.1 ± 25.7
Lee JT 2012 [16]	27	74.8 ± 6.5	19 M; 8F	–	AAA; failed EVAR	10.6 ± 7.5 (range:3–25)
Donas KP 2012 [17]	30	74.5 ± 7.3	27 M; 3F	–	AAA	15.2 ± 6.2
Coscas R 2011 [18]	13	74.8 ± 7.6	11 M; 2F	–	JAA	10.5 ± 5.5
Bruen KJ 2011 [19]	21	72 ± 8.0	11 M; 10F	–	JAA	12
Donas KP 2010 [20]	15	81.8	15 M; 0F	–	AAA	6.8 ± 3.3 (range:1–13)
Ullery BW [21]	60	75.8 ± 7.6	42 M; 18F	–	JAA; AAA; failed EVAR; Type IV TAAA	20.1 ± 21.0
**Total**	**430**	**74.5 ± 2.9**	**327 M; 93F; 10 undisclosed**	**–**	**–**	**16.5 ± 8.5**

*M* male, *F* female, *AAA* abdominal aortic aneurysm, *JAA* juxtarenal aortic aneurysm, *EVAR* endovascular aorta repair, *TAAA* thoraco-abdominal aortic aneurysm

### Aorta and main aortic endograft

Mean aortic landing zone length (proximal neck), defined as the infrarenal healthy aorta suitable for safe endograft apposition, measured 2.0 ± 2.0 mm, with an average diameter of 26.4 ± 4.3 mm (Table 2). The aortic endograft mean diameter was 26.4 ± 7.3 mm, and reported oversize was 19.5 ± 6.0%. In papers where the Nellix Endovascular Aneurysm Sealing System (Endologix Inc., Irvine, CA) was used to treat juxtarenal chronic aneurysm the diameter of the aortic endoprosthesis was not disclosed.

**Table 2:** Anatomic and technical details

Reference	Aortic neck length (mm)	Aortic neck diameter (mm)	Aortic endograft diameter (mm)	Aortic endograft oversizing (%)	Right renal artery diameter (mm)	Right CG diameter (mm)	Right CG oversizing (%)	Right CG length (mm)	Number of right CG implanted	Left renal artery diameter (mm)	Left CG diameter (mm)	Left CG oversizing (%)	Left CG length (mm)	Number of left CG implanted	Bilateral CG cases
Ullery BW 2017 [5]	1.1 ± 1.7	26.3 ± 3.6	–	–	–	6.2 ± 0.7	–	54.2 ± 8.9	15	–	6.2 ± 0.7	–	54.2 ± 8.9	12	–
Sugimoto M 2017 [6]	1.3 ± 2.7 2.7 ± 3.6	24.4 ± 3.0 25.5 ± 3.8	–	–	–	–	–	–	12	–	–	–	–	26	–
Caradu C 2017 [7]	1.8 ± 3.2	25.9 ± 2.8	30.1 ± 3.5	13.7 ± 4.7	5.3 ± 0.8	6.3 ± 0.5	18.9	51.4 ± 10.7	8	5.2 ± 1.0	6.5 ± 0.6	25	48.6 ± 12.4	20	–
Youssef M 2017 [8]	–	–	–	–	–	–	–	–	7	–	–	–	–	10	7
De Bruin JL 2016 [9]	1 (0–4)	33 (27–44)	–	–	–	–	–	–	21	–	–	–	–	18	11
Ronchey S 2015 [10]	–	–	29.5 ± 3.1	–	–	6.2 ± 0.8	25–30	48.2 ± 5.3	17	–	6.5 ± 0.8	25–30	55.3 ± 13.5	19	6
XiaoHui M 2015 [11]	7 ± 3.0	25 ± 4.0	–	–	–	5–7	–	20–80	21	–	5–7	–	20–80	35	14
Scali ST 2014 [12]	–	30.8	–	–	–	–	–	–	26	–	–	–	–	25	17
Ducasse E 2014 [13]	4.5 (1–9)	23 (18–32)	27.9 ± 3.5	15 (11–21)	–	5–7	–	36 ± 5.5	5	–	6.5 ± 0.6	–	37.6 ± 10.3	17	–
Tolenaar JL 2013 [14]	2.6 ± 3.2	27.1 ± 3.1	–	24.3 ± 7.4	6.6 ± 0.9	7 ± 1.0	13.9 ± 10.8	58.2 ± 20.9	10	6.4 ± 0.9	7 ± 0.9	14.6 ± 15.3	53.3 ± 8.9	9	7
Bin Jabr A 2013 [15]	–	–	13.5 ± 3.5	–	–	6.2 ± 0.4	–	20–40	4	–	6.3 ± 0.5	–	20–40	12	4
Lee JT 2012 [16]	1.4 ± 1.8	32.3 ± 6.3	31 ± 3.7	–	–	–	–	–	21	–	–	–	–	25	20
Donas KP 2012 [17]	–	–	–	–	5.5	6.3	14.5	–	19	5.5	6.3	14.5	–	16	5
Coscas R 2011 [18]	–	–	–	–	–	5–7	–	–	9	–	5–7	–	–	11	7
Bruen KJ 2011 [19]	0 (0–11)	27.1 ± 5.1	–	–	–	5–8	–	20–59	12	–	5–8	–	20–59	16	8
Donas KP 2010 [20]	0 (0–9)	16.4 ± 4.3	–	–	–	5.9 ± 0.25	–	27 ± 7.7	5	–	5.9 ± 0.25	–	27 ± 7.7	10	–
Ullery BW [21]	0.5 (0–9)	29.5 (15–58)	–	25 ± 6.6	–	–	–	–	53	–	–	–	–	44	33
**Total/Mean**	**2.0 ± 2.0**	**26.4 ± 4.3**	**26.4 ± 7.3**	**19.5 ± 6.0**	**5.8 ± 0.7**	**6.2 ± 0.3**	**18.1 ± 5.1**	**43.9 ± 11.1**	**265**	**5.7 ± 0.6**	**6.3 ± 0.3**	**19.1 ± 6**	**44 ± 10.7**	**325**	**139**

*CG* chimney graft

**Fig. 3:**
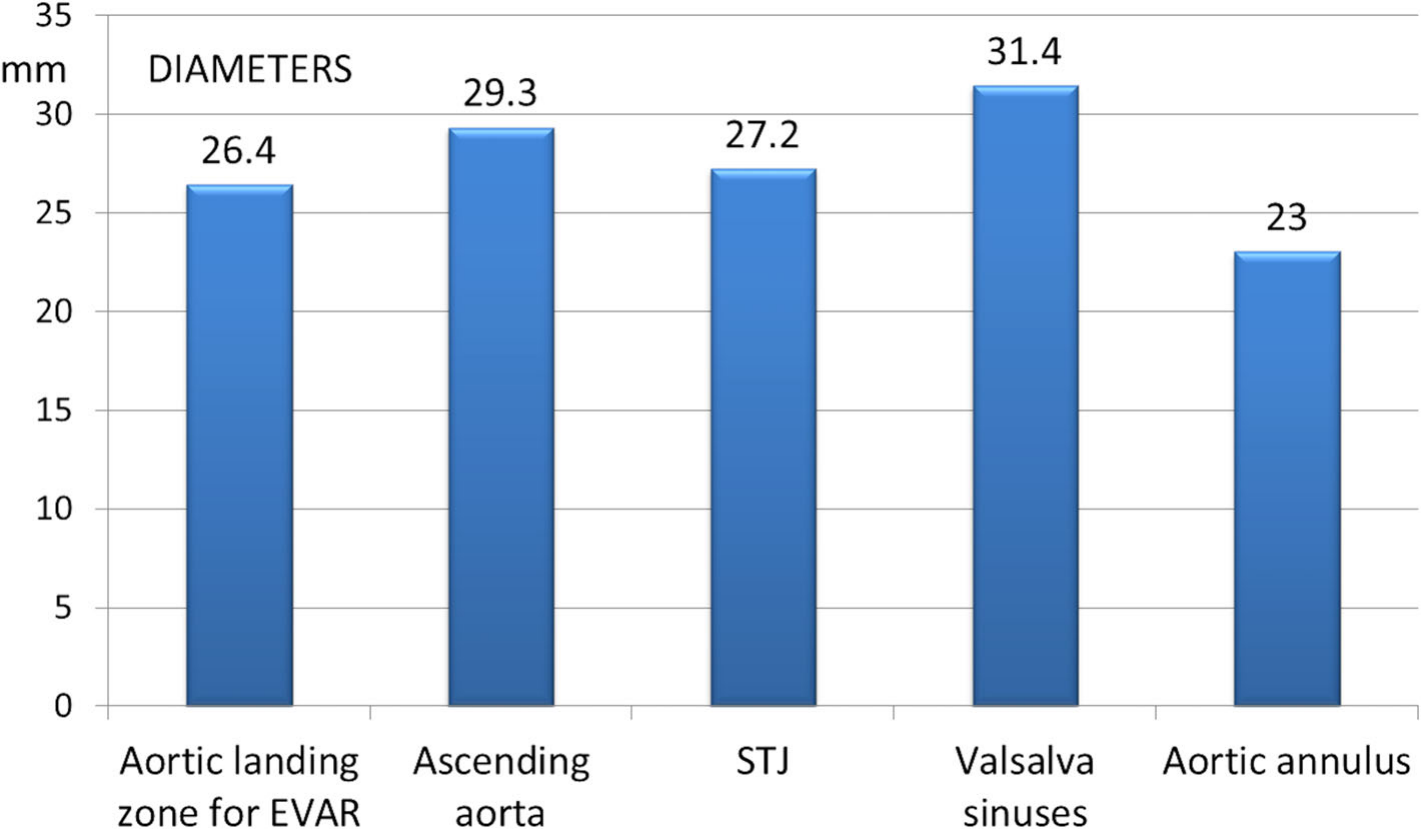
Comparison between proximal landing zone mean diameter of abdominal EVAR with renal CG and mean diameters of anatomic landmarks in ascending aorta and aortic root (published anthropometric data) (STJ = Sinotubular Junction)

In TARR, the distal landing zone is at the level of the distal ascending aorta, while the proximal landing zone is at the level of the aortic annulus and LVOT, including the aortic valve (the endograft presents a temporary valve). Standard anthropometric data show: ascending aorta mean diameter 3 cm above the sinotubular junction: 29.3 ± 4 mm; sinotubular junction mean diameter: 27.2 ± 3 mm; Valsalva Sinuses mean diameter: 31.4 ± 3.4 mm; aortic annulus mean diameter: 23 ± 2.5 mm (Fig. 3) [4]. These data show a correspondence between the diameter of the proximal lending zone of abdominal EVAR (26.4 ± 4.3 mm) and the diameter of the healthy distal ascending aorta (29.3 ± 4 mm) (11% bigger), the TARR device distal landing zone.

### Renal arteries and chimney grafts

Only three studies provide renal arteries diameters (Table 2). In two papers, right renal artery mean diameter was 5.3 ± 0.8 mm and 6.6 ± 0.9 mm, while the left was 5.2 ± 1.0 mm and 6.4 ± 0.9 mm. The third paper reported bilateral renal arteries mean diameter of 5.5 mm. Calculated left and right renal artery mean diameter is 5.7 ± 0.6 mm and 5.8 ± 0.7 mm, respectively.

**Fig. 4:**
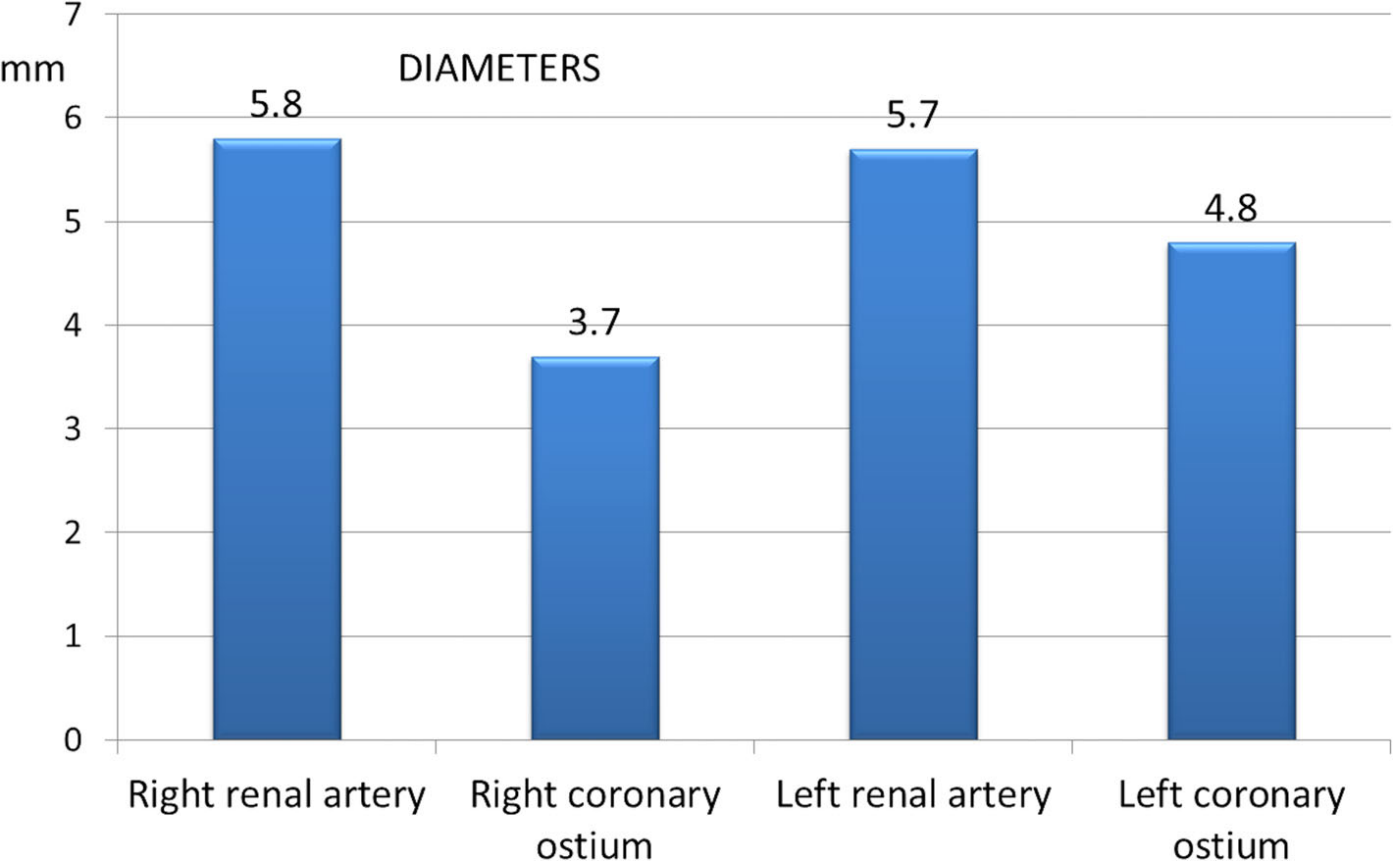
Comparison between mean renal artery diameters and mean coronary artery diameters (from published anthropometric data)

In Fig. 4, we compared renal arteries mean diameter with the mean diameter of non-diseased human coronary ostia taken from anthropometric data: the left renal artery is 19% bigger than the left coronary artery while the right renal artery is 56% bigger than the right coronary artery [4].

As to the renal CG, mean CG diameters were 6.2 ± 0.3 mm to the right and 6.3 ± 0.3 mm to the left with reported oversize of 18.1 ± 5.1% (right) and 19.7 ± 6% (left). Chimney graft mean length was 43.9 ± 11.1 mm (right) and 44 ± 10.7 mm (left). A total of 590 renal arteries were perfused with CG: 265 right and 325 left. In 139 patients (32.3%), bilateral renal CG were used.

**Fig. 5:**
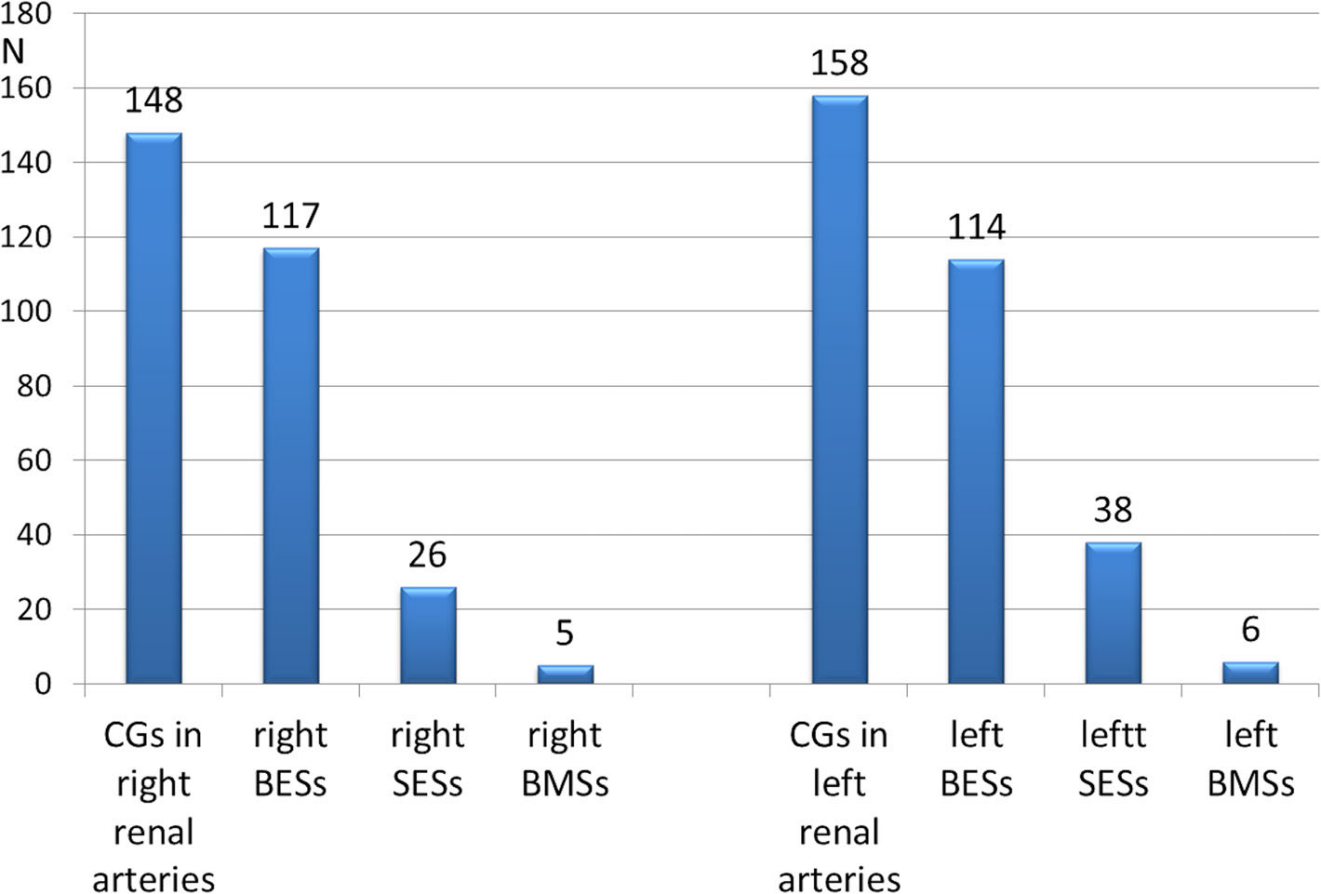
Number and type of renal CG. BESs = balloon-expanding covered stents; SESs = self-expanding covered stents; BMSs = balloon-expanding bare metal stents

If we look at CG type, we have data of 306 CG (62%) (Fig. 5). Among CG implanted in right renal arteries, 117 were balloon-expanding covered stents (BESs), 26 were self-expanding covered stents (SESs), and 5 were balloon-expanding bare metal stents (BMSs). In left-sided CG, numbers were 114, 38 and 6, respectively. In total, 46.8% (*n* = 231) were BESs, 13% (*n* = 64) were SESs, 2.2% (*n* = 11) were BMSs, 38% were undisclosed.

### Complications

Nineteen occlusions were reported (3.2%) but there was no information about the CG occlusion rate on each side. The shortest period between implant and occlusion was 2 days. Reported treatments included thrombectomy followed by renal bypass and dialysis for stage-3 kidney failure (Table 3). Endoleaks were reported in 83 patients (19.3%): 67 (15.6%) early and 22 (5.9%) late endoleaks (Table 3). Early EL consisted of 40 EL-I, 24 EL-II, 3 EL-III, while late EL were 10 EL-I, 9 EL-II, 3 undetermined. Most EL were managed conservatively but seven patients underwent reinterventions for proximal extension (5 EL-I) or embolization (1 EL-I, 1 EL-II).

**Table 3:** Outcome of renal chimney grafts

Reference	Patients	CG occluded	Timing	Treatment	Endoleak < 30 days	Endoleak > 30 days	Treatment
Ullery BW 2017 [5]	15	2	–	no intervention	5EL-Ia, 3 EL-II	–	proximal extension for 3 EL-Ia
Sugimoto M 2017 [6]	38	2	1 at 22 months 1 at 24 months	–	1 EL-Ia	–	–
Caradu C 2017 [7]	26	2	1 at< 30 days; 1 late	–	2 EL-I; 4 EL-II	–	–
Youssef M 2017 [8]	10	0	–	–	0	0	–
De Bruin JL 2016 [9]	28	0	–	–	–	1 EL-Ia; 1 EL-II	Coil embolization of EL-Ia
Ronchey S 2015 [10]	20	1	at 11 months	no intervention	0	0	–
XiaoHui M 2015 [11]	42	1	at 3 months	–	8 EL-Ia	5 EL-Ia	–
Scali ST 2014 [12]	41	6	1 at 40.4 months 1 at 40.4 months 1 at 2.1 months 1 at 11.5 months 2 < 30 days	1 renal bypass	–	3 EL-Ia; 4 EL-II; 3 undetermined	–
Ducasse E 2014 [13]	22	0	–	–	1 EL-Ia	–	–
Tolenaar JL 2013 [14]	12	2	1 < 2 days; 1 late	no intervention	–	1 EL-II	Coil embolization of EL-II
Bin Jabr A 2013 [15]	10	0	–	–	–	–	–
Lee JT 2012 [16]	27	1	at 3 months	–	2 EL-I; 2 EL-II; 3 EL-III	–	proximal extension for 1 EL-III
Donas KP 2012 [17]	30	1	at 45 days	Open renal artery thrombectomy and ileo-renal bypass	–	2 EL-II	–
Coscas R 2011 [18]	13	0	–	–	2 EL-Ia	1 EL-Ia	–
Bruen KJ 2011 [19]	21	0	–	–	1 EL-Ia; 2 EL-II	1 EL-II	–
Donas KP 2010 [20]	15	1	at 45 days	Open renal artery thrombectomy and ileo-renal bypass	1 EL-II	–	–
Ullery BW [21]	60	0	–	–	18 EL-Ia; 12 EL-II	–	proximal extension for 1 EL-Ia
**Total**	**430**	**19 (3.7%)**	**–**	**3 procedures**	**40 EL-I** **24 EL-II** **3 EL-III**	**10 EL-Ia** **9 EL-II** **3 undetermined**	**7 procedures**

*CG* chimney graft, *EL* endoleak

## Discussion

Increasing data support the use of CG to preserve visceral organs during EVAR [5–24]. Recently, Usai et al. presented a systematic review on frequency and clinical relevance of CG occlusions: among 8 suitable studies with mean follow-up of 15.6 months, authors reported a 4.7% incidence of CG occlusion [25]. Similarly, Nordon et al., in an earlier review, showed six-month patency rate of 97.7%, with presence of late endoleak in 17 patients (9.7%) and reinterventions in 5 (2.8%) [26]. In accordance, our review shows CG occlusion rate of 3.7% and EL in 19.3%, confirming that CG is feasible and safe in selected EVAR cases.

Should we therefore consider this technique a clinical model for coronary perfusion in TARR techniques? In order to maintain the focus on the safety of our patients, some very important considerations are required before any attempt to reproduce this technique in human beings will be pursuit by using newly developed or available devices.

The choice for appropriate main endograft diameter and shape is important to prevent type-I endoleak at proximal and distal landing zones. Lachat et al., suggested an elliptic model for the estimation of the size: diameter should be based on aorta diameter at landing zone plus the diameter of the CG [27]. Another rule is to empirically oversize the main graft by 20–30% [28]. When we analysed published data, mean diameter at landing zone was 26.4 mm and mean reported oversize was 19.5% (data limited to 4 reports). If we speculate that TARR device will follow the same rules, for a mean distal ascending aorta diameter of 29.3 mm (distal landing zone in anthropometric data) we should implant 35-38 mm diameter endografts (20–30% oversizing). In a previous report of endovascular aortic procedures in non-dissected ascending aorta, endografts mean diameter was 36.6 ± 6.5 mm, reflecting a good accordance with general vascular behave [1]. With regards to the proximal lending zone at the level of aortic annulus and LVOT, the sizing will follow the same rules developed for the transcatheter aortic valve replacement. This will allow safe deployment of the TARR main endograft that should include, on proximal side, a temporary non-biological aortic valve. A standard transcatheter valve will then be placed, transfemorally or gransapically, into the endoprosthesis and deployed at the level of the temporary valve.

As for coronary arteries, there are similarities between coronary and renal artery anatomy and, therefore, CG can potentially be used for myocardial perfusion in TARR. Some considerations are required before using little-diameter endografts in small arteries. Anthropometric data show average coronary ostia diameters of 4.8 mm (left) and 3.7 mm (right), while this review shows left and right renal artery diameters of 5.7 mm and 5.8 mm, respectively, with CG diameters of 6.3 mm (left) and 6.2 mm (right) [4]. Therefore, we can speculate that coronary CG of 5 mm (left) and 4 mm (right) will oversize of approximately 20% the coronary ostia and will be effective in maintaining the coronary flow. Double antiplatelet therapy should be used to prevent CG thrombosis that would determine severe myocardial damages, while the curved aortic wall should prevent CG kinking. In order to better explore the anatomical relationships of the dilated root and the coronaries we have already tested the possibility of developing high-fidelity 3D-printed root models from CT-scans that could also be used to test the new devices and the coronary flows [29]. Nevertheless, further tests are required to better understand some challenges: 1) parallel grafts in pararenal aortic segment have wall contact while in the Valsalva Sinuses they have more degrees of freedom and, potentially, dislodgement; 2) movements of the root caused by the beating heart can affect the CG stability; 3) the length of the coronary left main stem can vary significantly in the population.

Another important point in coronary CG is the length of endografts. In the setting of a device for root disease treatment, the main endoprosthesis has to be rather long in order to reach enough fixation at the level of the non-diseased aorta: the endograft will easily be extended from LVOT to the distal ascending aorta and therefore coronary CG will be longer than renal CG: instead of 4-5 cm long, the coronary CG will easily be 10 cm long. Increased length will increase the risk of CG thrombosis but, so far, studies reporting the associations between CG length and risk of occlusion are lacking and research about risk factors for CG failure are rare. Pecoraro et al., suggested that incomplete expansion, inadequate length, and small diseased target arteries are risk factors for occlusion, as well as an oversize of less than 20%, as suggested by Tanious et al. [28, 30]. Therefore, use of CG in coronaries should be suggested only for patients with adequate coronary ostia diameter and absence of atherosclerotic disease in the first part of the left main and the right coronary artery (1–1.5 cm). Additionally, coronary perfusion will be mainly guarantee by diastolic blood flow through the distal CG orifices that are placed far from the original ostia: this fact can potentially cause a drop in coronary bloodstream leading to adverse consequences.

Endoleaks are important. Ullery et al., reported that long-term anticoagulation, oversize degree, stent type/diameter, and other clinical/anatomical variables were not significantly associated with endoleaks in EVAR [21]. In their research, 60 patients required 97 renal CG (33 bilateral), 12 superior mesenteric CG, and 2 celiac artery CG. Early gutter-related type-Ia endoleaks were noted in 30% of postoperative imaging studies but the follow-up revealed spontaneous resolution in 44.3, 65.2, and 88.4% of cases at 6, 12, and 18 months postoperatively. Therefore, same results can be foreseen in TARR techniques or different endograft shapes can be developed to better adapt the CG to the main endoprosthesis.

Another transcatheter technique to address the dilated ascending aorta was recently described but the prototype doesn’t cover the root and, therefore, there was no need for development of new strategies for coronary perfusion [31].

### Limits of the study

This is a review of published renal CG reports and there could be a risk of publication bias because several data are missing and there is a degree of heterogeneity in the type of aortic diseased described. In addition, lack of standardization in CG technique and devices provides us with a mixture of different endoprosthesis and chimney grafts. Moreover, on purpose we only analysed the chimney technique but other options such as endograft fenestration, short interruption or periscope-graft could be taken in consideration in view of new TARR devices.

## Conclusion

Renal CG during EVAR is feasible in selected cases and coronary CG for TARR can be the object of further studies. However, relevant points of difference should be taken into consideration: coronary ostia are smaller than renal arteries (particularly the right) and coronary grafts would be longer than renal CG, with a higher risk of thrombosis.

## Data Availability

Data are available in PubMed (see references).
